# Optimization of curdlan production and ultrasound assisted extraction processes from *Priestia megaterium*

**DOI:** 10.1038/s41598-024-77880-y

**Published:** 2024-11-04

**Authors:** Natasha Aquinas, Bhat M. Ramananda, Subbalaxmi Selvaraj

**Affiliations:** Department of Biotechnology, Manipal Institute of Technology, Manipal Academy of Higher Education (MAHE), Manipal, 576104 Karnataka India

**Keywords:** Curdlan, Optimization, Extraction, Ultrasonication, *Priestia megaterium*, Industrial microbiology, Metabolic engineering

## Abstract

In this study, the upstream and downstream production processes of curdlan from *Priestia megaterium* were optimized to enhance its yield. Additionally, a novel extraction method was developed for curdlan recovery. Optimization studies were conducted using Central composite design (CCD). Curdlan yield improved from 0.15 g/L (unoptimized) to 0.46 g/L (3-fold increase) when fermentation was carried out in CCD-optimized media of (w/v) sucrose 20%, urea 0.1%, KH_2_PO_4_ 0.02%, agitation speed 250 rpm. To further enhance curdlan yield during extraction, ultrasonication was incorporated as a novel step into the conventional method of acid/alkali-assisted curdlan recovery. A two-step optimization was chosen for extraction, namely, one-factor-at-a-time (OFAT) and CCD, wherein the optimized extraction parameters were determined to be 25 s sonication, 1 N NaOH, and 2 h solubilization time. The curdlan yield improved by 1.5-fold (0.70 g/L) post optimization, in comparison with unoptimized conventional extraction step. Finally, the biopolymer was validated through characterization by nuclear magnetic resonance (NMR) which showed characteristic curdlan spectra in the^13^C and^1^H NMR studies. To the best of our knowledge, this study represents the first documented report on curdlan extraction using this novel method of ultrasonication.

**Supplementary Information:**

The online version contains supplementary material available at 10.1038/s41598-024-77880-y.

## Introduction

Curdlan is a water insoluble exopolysaccharide which consists of glucose units linked together by β-1,3-glycosidic linkages^1^. It is a neutral polysaccharide that has been utilized in both the food and pharmaceutical industries due to its remarkable thermal and gelling properties^2^. Curdlan is capable of forming two types of gels based on the temperature at which its aqueous solution is heated. The thermo-reversible or low-set gel is formed when aqueous solution of curdlan is heated to about 55 °C and the thermo-irreversible gel is formed at around 85 °C. This occurs because its structure can form condensed rod-like triple helices when subjected to heating at higher temperatures^3^. Some bacteria that have been reported to produce curdlan include *Agrobacterium* sp., *Rhizobium* sp., *Bacillus* sp., *Pseudomonas* sp., *Cellulomonas* sp^4–8^. Because it is a biopolymer, curdlan is deemed to be non-toxic, biodegradable, and possesses good biocompatibility^2^. Curdlan has been utilized in Japan and Korea since 1989, and it received approval from the U.S. Food and Drug Administration (FDA) for use in the food industry in 1996 ^9^.

Curdlan tends to stand out when compared with other gums like gelatin, agar, carrageenan due to its wider range of applications and distinctive physicochemical properties. Curdlan has a better gelation strength at higher temperatures as against the other polymers^10^. Curdlan has found applications in the food industry in products such as noodles, sauces, jellies, frozen food, and packaged meat^11^. This is possible due to its thermal gelling and exemplary rheological properties. These properties of curdlan also aid in improving food texture and provide stability^12^. Curdlan, being a β-glucan, can elicit an immune response through recognition by macrophages and other immune cells^13^. In the biomedical sector, it has been studied as a drug-delivery carrier owing to its capability to sustain and control drug release. Modified forms of curdlan such as curdlan-sulfate, curdlan hydroxyethyl derivatives and carboxymethylated curdlan have also shown promising results in the biomedical and pharmaceutical industry^14,15^. Some of these derivatives have been applied in as anti-AIDS agents to help in fighting HIV infections^16^. The gel forming property of curdlan has led to growing interest towards curdlan hydrogels. Several studies have demonstrated that curdlan hydrogels could be potentially utilized in oral, dental, and transdermal drug delivery^17^.

Commercial production of curdlan is carried out on a large-scale using *Agrobacterium* sp. ATCC 31,749 and *Agrobacterium* sp. ATCC 31,750 since both strains are known to produce high yields of curdlan by fermentation, capable of utilizing different types of carbon sources. A notable curdlan yield of 60 g/L was achieved by *Agrobacterium* sp. ATCC 31,750 when sucrose was used as the carbon source, diammonium hydrogen phosphate as the nitrogen source, with a fermentation time of 115 h. In contrast, *Agrobacterium* sp. ATCC 31,749 produced 48 g/L curdlan using sucrose as carbon source, and yeast extract as nitrogen source at 168 h of fermentation^18,19^. However, the challenges in terms of cost-effectiveness for researchers, particularly in countries where it is not readily available also needs to be considered. They will have to rely on importing commercial curdlan from other countries such as Japan, USA, or China. A potential solution to this is to domestically explore alternate sources for curdlan and optimize its production. An additional approach involves optimizing not just the upstream production, but also the extraction of curdlan. In the case of other biopolymers such as polyhydroxyalkanoates (PHA), mechanical disruption of cells has been used for as it is a intracellular biopolymer. This can be carried out through various methods such as ultrasonic disruption bead mills and high-pressure homogenization^20^. The precipitation method, involving alkali and acid neutralization, is a widely used and cost-effective approach for curdlan extraction. This method is not only easy to execute but also offers opportunities for yield enhancement through slight modifications which has been explored in this study. Treatments combining alkaline conditions with ultrasonication have been shown to enhance product yield by improving solubility, emulsification, and gelation properties^21^. Therefore, in this study, the conventional precipitation method was modified to include an ultrasonication step to enhance its release, curdlan being a cell-bound extracellular biopolymer. Previous studies have focused mainly on optimizing the media components and pH^11,14,22,23^. Hence, we aimed to optimize the fermentative production of curdlan by previously isolated *Priestia megaterium*^24^ through response surface methodology (RSM) and further develop a novel method of ultrasonication for extraction of curdlan. The extraction of curdlan was also optimized by one-factor-at-a-time (OFAT) and RSM to achieve higher yields of curdlan. Finally, the structural characteristics of curdlan were elucidated by Nuclear magnetic resonance (NMR). This analytical technique provided detailed insights into the molecular configuration of the biopolymer.

## Materials and methods

### Materials

All media components, chemicals and reagents used in the study were of analytical grade purchased from HiMedia, Sigma-Aldrich, and Loba.

### Strain and inoculum preparation

Curdlan producing *Priestia megaterium* employed in the current study was originally isolated from cow pea soil as described in prior investigation^24^. Culture inoculum was prepared by cultivating *P. megaterium* in a growth medium consisting of 2% (w/v) sucrose, 0.5% (w/v) peptone, 0.5% (w/v) yeast extract at pH 7.0, 30 °C, 180 rpm for 18 h, with inoculum density corresponding to 0.5 McFarland standard. After this incubation period, 10% of the inoculum was transferred to the fermentation media.

### Submerged fermentation

The fermentation media was prepared in a 250 mL Erlenmeyer flask containing 100 mL of working volume. Based on the preliminary studies^24^, the media consisted of (% w/v) sucrose (15), urea (0.1), KH_2_PO_4_ (0.1), MgSO_4_.7H_2_O (0.04) and 1% trace elements solution in 0.1 N HCl (g/L): FeSO_4_.7H_2_O (5), MnSO_4_.H_2_O (2), CoCl_2_.6H_2_O (1), ZnCl_2_ (1). The pH, incubation temperature, inoculum size and agitation speed were kept at 7.0, 30 °C, 10%, and 180 rpm, respectively. The fermentation media was incubated for 96 h^24^.

### Optimization of media and process parameters by RSM using central composite design (CCD)

The preliminary optimization for fermentative production of curdlan was carried out by OFAT method in our previous study^24^. Media components such as sucrose, urea, KH_2_PO_4_, MgSO_4_.7H_2_O and physical parameters such as pH, inoculum size, agitation speed were optimized using the OFAT approach^24^.

For further optimization, RSM by CCD was employed. RSM is used for testing several parameters as fewer experimental runs are needed as compared to OFAT^14^. From OFAT studies, it was noted that the major factors that influence the yield of curdlan were sucrose, urea, KH_2_PO_4_, and agitation speed^24^. Therefore, these four factors were chosen for RSM studies. The variables and their coded levels (-2, -1, 0, + 1, +2) are shown in Table 1.

**Table 1:** Fermentation variables and their levels for CCD.

Variables and their codes	Levels				
	-2	-1	0	+ 1	+ 2
A = Sucrose %	4	8	12	16	20
B = Urea %	0.05	0.1	0.15	0.20	0.25
C = KH_2_PO_4_%	0.02	0.06	0.1	0.14	0.18
D = Agitation (rpm)	50	100	150	200	250

For four factors, this trial was a full 2^4^ factorial design with eight axial or star points and six replications of the centre point (level 0). A total of 30 experiments were generated for four factors. The distance between the star points and the centre point was given by α = 2^n/4^ (for 4 factors *n* = 4, α = 2). The quality of fit of the model was given by the coefficient of determination R^2^ and the statistical fitness of the model was given by analysis of variance (ANOVA). The statistical software used for analysis was Design-Expert version 9 (Stat-Ease, Minneapolis, MN, USA).

### Extraction of curdlan from the fermentation media

The conventional method of curdlan extraction from the fermentation media uses HCl and NaOH precipitation. In this method, the fermentation media was first centrifuged at 10,000 rpm for 15 min at 4 °C. The pellet obtained was solubilized in 2 N NaOH overnight at 30 °C in a rotary shaker. Post solubilization, centrifugation was again carried out at 10,000 rpm for 15 min at 4 °C and 2 N HCl was added to the resultant supernatant to precipitate out the curdlan. The curdlan precipitate was rinsed with distilled water and then dried in a hot air oven at 60 °C for 7 to 8 h. The dried precipitate was weighed to determine the curdlan yield^24,25^.

**Fig. 1:**
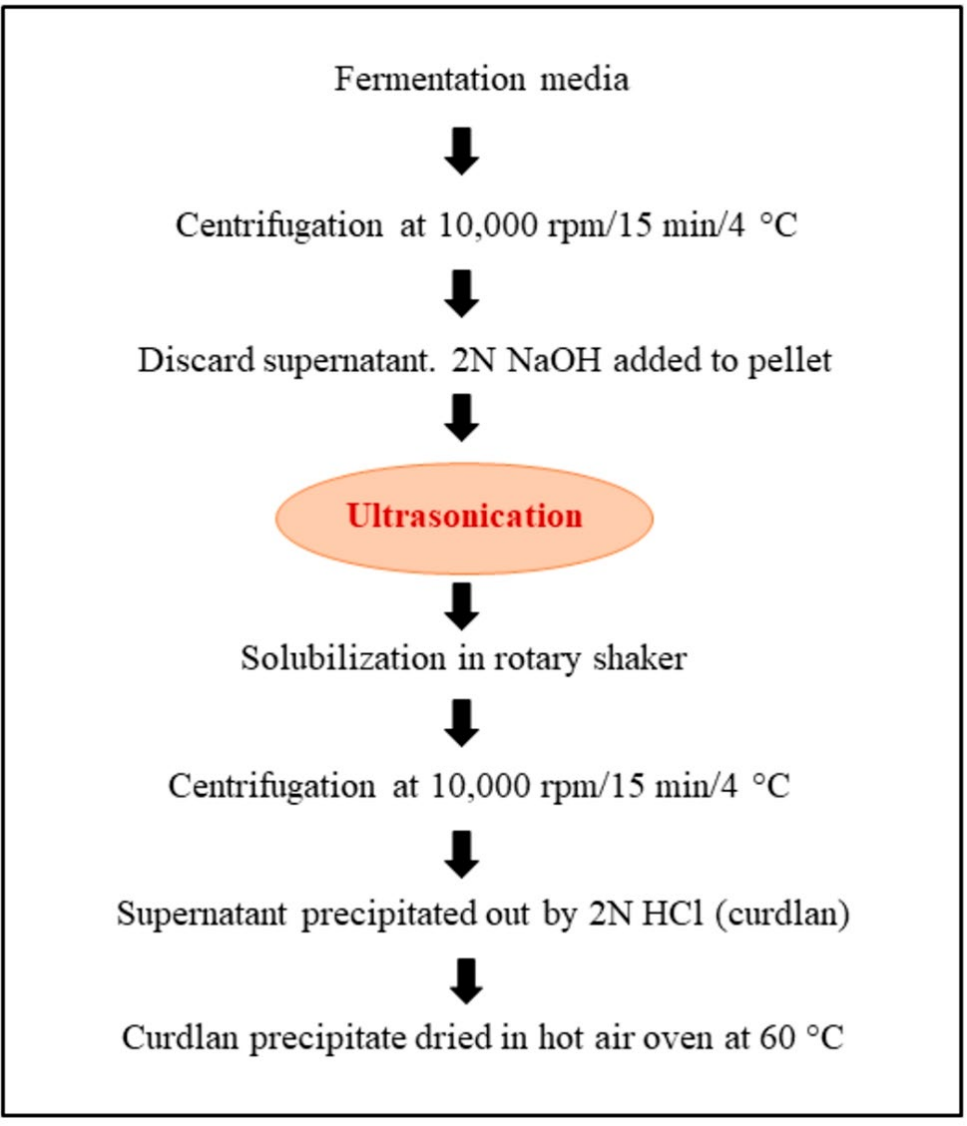
Ultrasound-assisted extraction of curdlan produced by *Priestia megaterium*.

Ultrasound-assisted extraction of polysaccharides has recently gained prominence due to its higher extraction efficiency and reducing processing time^26^. The mechanism of ultrasound-assisted extraction relies on the ability to produce cavitation along with mechanical and thermal changes. It has also been reported that ultrasound-assisted extraction improves the rate of extraction of polysaccharides^27^. Therefore, to improve the yield of curdlan, ultrasonication was carried out. The ultrasonication step was introduced after the addition of NaOH and both bath and probe sonications were utilized for this purpose. Bath sonication generates ultrasonic waves of lower energy density in comparison with Probe sonication. Hence, the authors screened both techniques to increase the yield of curdlan during extraction. Bath sonication was carried out in the Biobee bath sonicator instrument (LMUC-3), where the ultrasound conditions were set at 100 W, 40 kHz frequency, and at room temperature, since considerable increase in temperature is not observed during the process. Sonication was carried out for different time periods of 30s, 1, 2, 3, 4, 5 min. For probe sonication, the Sonics Vibracell instrument (VCX 130) was used. The ultrasound conditions were set at 130 W, 20 kHz frequency, 60% amplitude, pulse on, and time duration of 5, 10, 20, 30, 40, 50 s. The entire process was carried out in cold conditions in an ice bath (~ 0°) to prevent heating of cell suspension due to extremely high energy density. Following ultrasonication, the extraction process was continued as usual as per the conventional method. A process flow chart showing the steps in ultrasound-assisted curdlan extraction is given in Fig. 1.

### Optimization of extraction of curdlan using ultrasonication

The overall cost can be reduced by enhancing productivity. Typically, higher productivity can be achieved either through strain improvement or by process parameter optimization. However, this presents a ‘catch-22’ situation wherein selecting a lead strain requires an optimal medium and identifying the best medium requires a lead strain^28^. To overcome this limitation, newer strategies such as downstream processing optimization are needed for yield improvement. Optimizing the extraction of curdlan helps in identifying the optimal conditions at which maximum yield is obtained and improving efficiency. Two step optimization has been used in this study viz. OFAT and CCD to screen the parameters as well as study their interaction effects, respectively.

#### One-factor-at-a-time (OFAT) method

To determine the optimal level of extraction parameters, optimization by OFAT was carried out. The sonication time (s), NaOH concentrations, and solubilization time (h) were varied at a time keeping the other factors constant. Time periods chosen for solubilization were 2, 4, 8, 12, 16, 20, 24 h and different NaOH concentrations chosen were 1, 1.5, 2, 2.5, 3 N. The yield of curdlan was determined at all these different levels by drying the resultant precipitate in a hot air oven at 60 °C.

#### Central composite design (CCD)

The central composite design (CCD) of RSM was employed to further optimize the parameters affecting curdlan extraction. The variables used were sonication time (s), NaOH concentration, and solubilization time (h). The variables and their coded levels (-2, -1, 0, + 1, +2) are shown in Table 2.

**Table 2:** Extraction variables and their levels for CCD.

Variables and their codes	Levels				
	-2	-1	0	+ 1	+ 2
A = NaOH (N)	1	1.5	2	2.5	3
B = Solubilization time (h)	2	4	6	8	10
C = Sonication time (s)	5	10	15	20	25

For three factors, this trial was a full 2^3^ factorial design with six axial or star points and six replications of the centre point (level 0). A total of 20 experiments were generated for three factors. The distance between the star points and the centre point was given by α = 2^n/4^ (for 3 factors *n* = 3, α = 1.68). The quality of fit of the model was assessed using the coefficient of determination R^2^ while the statistical fitness of the model was evaluated through analysis of variance (ANOVA). The statistical software used for analysis was Design-Expert version 9 (Stat-Ease, Minneapolis, MN, USA).

### Statistical analysis

Data are represented as the mean ± standard deviation (SD) of three replicates. Statistical analyses were performed using Microsoft Excel 2016.

### Characterization of curdlan

Nuclear magnetic resonance (NMR) analysis of curdlan was carried out to analyse the structure of curdlan^1^. H NMR and^13^C NMR spectroscopy were performed using the Bruker Ascend 400 MHz NMR Instrument. The lyophilized curdlan sample was dissolved in dimethyl sulfoxide (DMSO) for the analysis.

## Results and discussion

### Optimization of media and process parameters by RSM using CCD

Based on our previous studies^24^, CCD was designed to determine the optimal levels and the interaction effects of the factors in the production of curdlan. The results of the experimental curdlan production by four factors are shown in Table 3. The maximum yield of curdlan production was found to be 0.43 g/L from run number 16 where the parameters were (w/v): sucrose 16%, urea 0.1%, KH_2_PO_4_ 0.06%, and 200 rpm. The minimal variation among the central points suggests that the experimental data exhibits notable reproducibility. The fitness of the model was given by ANOVA as shown in Table 4. Whereas our previous study showed the curdlan yield of 0.35 g/L with traditional OFAT method^24^.

**Table 3:** Central composite design for curdlan production with observed and predicted data.

Run no.	A: Sucrose %	B: Urea %	C: KH_2_PO_4_%	D: Agitation (rpm)	Curdlan yield (g/L) Actual	Curdlan yield (g/L) Predicted
1	12	0.05	0.1	150	0.12	0.137
2	8	0.1	0.14	200	0.25	0.224
3	8	0.1	0.06	100	0.09	0.091
4	16	0.1	0.14	200	0.28	0.305
5	4	0.15	0.1	150	0.07	0.057
6	8	0.2	0.06	100	0.001	0.003
7	12	0.15	0.1	150	0.18	0.183
8	12	0.15	0.1	250	0.31	0.354
9	16	0.2	0.06	100	0.03	0.022
10	8	0.2	0.06	200	0.11	0.085
11	16	0.2	0.14	100	0.06	0.078
12	8	0.1	0.14	100	0.135	0.150
13	8	0.1	0.06	200	0.19	0.193
14	16	0.2	0.14	200	0.38	0.345
15	12	0.25	0.1	150	0.09	0.083
16	16	0.1	0.06	200	0.43	0.363
17	16	0.1	0.06	100	0.05	0.057
18	12	0.15	0.1	150	0.2	0.183
19	12	0.15	0.18	150	0.33	0.304
20	12	0.15	0.1	50	0.02	0.013
21	12	0.15	0.1	150	0.17	0.183
22	16	0.2	0.06	200	0.31	0.316
23	20	0.15	0.1	150	0.14	0.163
24	16	0.1	0.14	100	0.035	0.026
25	12	0.15	0.1	150	0.19	0.183
26	12	0.15	0.02	150	0.18	0.216
27	12	0.15	0.1	150	0.18	0.183
28	12	0.15	0.1	150	0.18	0.183
29	8	0.2	0.14	100	0.11	0.143
30	8	0.2	0.14	200	0.19	0.204

**Table 4:** ANOVA for Curdlan yield.

Source	Sum of squares	df	Mean square	F-value	*p*-value
Model	0.3309	14	0.0236	21.71	< 0.0001^*^
A-Sucrose	0.0170	1	0.0170	15.63	0.0013^*^
B-Urea	0.0045	1	0.0045	4.14	0.0599
C-KH_2_PO_4_	0.0117	1	0.0117	10.71	0.0051^*^
D-Agitation	0.2033	1	0.2033	186.76	< 0.0001^*^
AB	0.0036	1	0.0036	3.28	0.0902
AC	0.0081	1	0.0081	7.40	0.0158^*^
AD	0.0421	1	0.0421	38.70	< 0.0001^*^
BC	0.0076	1	0.0076	6.99	0.0184^*^
BD	0.0002	1	0.0002	0.1493	0.7046
CD	0.0007	1	0.0007	0.6821	0.4218
A^2^	0.0091	1	0.0091	8.36	0.0112^*^
B^2^	0.0091	1	0.0091	8.36	0.0112^*^
C^2^	0.0102	1	0.0102	9.37	0.0079^*^
D^2^	0.0003	1	0.0003	0.2610	0.6169
Residual	0.0163	15	0.0011		
Lack of fit	0.0158	10	0.0016	14.81	0.0041^*^
Pure error	0.0005	5	0.0001		
Cor Total	0.3472	29			

*Represents statistically significant at 95% probability.

The model’s F value of 21.71, as displayed in Table 4, suggests that the model holds statistical significance. The model fits the data better as the F value of model (21.71) is higher than the F value of lack of fit (14.81). The F value for the lack of fit may be significantly large from the existence of noise in the data. The coefficient of variation (C.V. %) was 19.75 demonstrating good reproducibility (C.V. % <10% denotes excellent, 10–20% denotes good, 20–30% denotes adequate, and > 30% denotes poor reproducibility^29^), while the adequate precision value of 16.18 which indicated an adequate signal, as a ratio of more than 4 is considered desirable. The pure error is exceptionally low, indicating a high reproducibility of the obtained data. At a confidence level of 95% and *p* < 0.05, the linear terms A and D in the current model are statistically significant. A smaller p-value has greater influence on the yield and values < 0.05 are said to be statistically significant^30^. Among the two-way interactions, the AC, AD, BC terms are significant and among the squared interactions, the A^2^, B^2^, C^2^ terms are significant. The coefficient of determination R^2^ quantifies the extent to which the predicted values accurately reflect the experimental values, thereby demonstrating the degree of correlation between them^31^. The R^2^ for the correlation between the predicted and observed values for curdlan yield was 0.95. The model can explain up to 95% variation in the response.These results were compared with previous RSM studies on optimization of curdlan yield from *Pseudomonas* sp., *Agrobacterium* sp., and *Paenibacillus polymyxa* ATCC 21,830. The R^2^ value of curdlan optimization from *Pseudomonas* sp. was reported as 0.82, where the curdlan yield was 2.35 g/L at optimized conditions of glucose 103 g/L, pH 7.6, and yeast extract 1.52 g/L at 200 rpm for 72 h^22^. In the case of *Agrobacterium* sp., a yield of 5.02 g/L at optimized conditions of sucrose 142.9 g/L, urea 0.52 g/L, pH 7.5 at 250 rpm for 120 h was achieved, where the R^2^ value was 0.81 ^14^. The curdlan yield reported for *Paenibacillus polymyxa* ATCC 21,830 was 6.89 g/L with R^2^ value of 0.87. The optimum culture conditions were glucose 100 g/L, yeast extract 3 g/L, pH 7 at 150 rpm for 96 h^11^. The model was modified by removing the insignificant terms. However, since the fitness of the model was better in the current model, hence the model is retained (Eq. 1). Results of the modified model are shown in the supplementary files for further information (Table S1).

The regression equation for the model in coded units is given in Eq. 1:

1 $$\begin{aligned} {\text{Y}}_{{\left( {{\text{g}}/{\text{L}}} \right)}} = & 0.{\text{1833 }} + {\text{ }}0.0{\text{266A }} - {\text{ }}0.0{\text{137B }} + {\text{ }}0.0{\text{22}}0{\text{C }} + {\text{ }}0.0{\text{92}}0{\text{D }} \\ & \; + {\text{ }}0.0{\text{149AB }} - {\text{ }}0.0{\text{224AC }} + {\text{ }}0.0{\text{513AD }} + {\text{ }}0.0{\text{218BC }} - {\text{ }}0.00{\text{32BD}} \\ & \; - {\text{ }}0.00{\text{68CD }} - {\text{ }}0.0{\text{182A}}^{{\text{2}}} - {\text{ }}0.0{\text{182B}}^{{\text{2}}} + {\text{ }}0.0{\text{193C}}^{{\text{2}}} - 0.00{\text{32D}}^{{\text{2}}} \\ \end{aligned}$$

Where Y is the curdlan yield and A, B, C, D are sucrose, urea, KH_2_PO_4_, agitation, respectively.

**Fig. 2:**
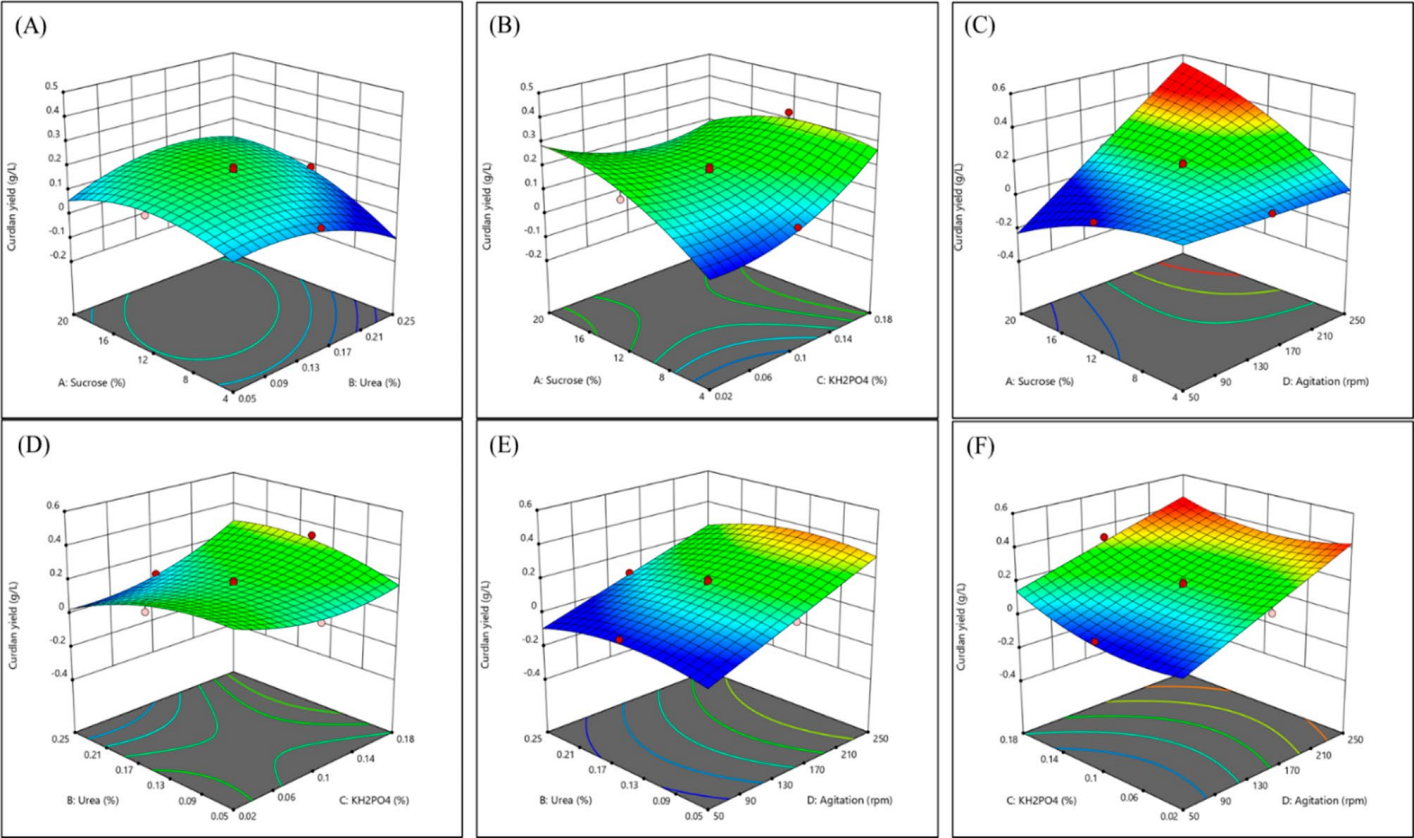
Response surface plot showing effect of different factors on curdlan yield. (**A**) sucrose and urea; (**B**) sucrose and KH_2_PO_4_; (**C**) sucrose and agitation; (**D**) urea and KH_2_PO_4_; (**E**) urea and agitation; (**F**) KH_2_PO_4_ and agitation. (Generated by Design-Expert software, version 9)

3-D surface plots of curdlan yield and the factors were generated (Fig. 2) illustrating the dependency of curdlan yield on these factors. With increased urea and decreased sucrose concentration, curdlan yield decreased (Fig. 2A). This likely results from the release of excess ammonium ions, which exerts an inhibitory effect on curdlan production^25^. However, at higher sucrose and lower KH_2_PO_4_ concentrations, it was seen that the curdlan production slightly increased (Fig. 2B). KH_2_PO_4_ is essential for fundamental cellular processes. Hence, reduced concentrations of KH_2_PO_4_ likely aid in sustaining cell growth and curdlan production without inducing inhibitory effects from excess phosphate ions^32^. Furthermore, it is observed that at increased sucrose concentration and higher agitation speed, the yield of curdlan greatly increased (Fig. 2C). Agitation speed is essential for aeration and oxygen supply^24^. This also correlates with the improved curdlan yield with higher agitation speed. Decreased urea and KH_2_PO_4_ concentrations improved curdlan yield (Fig. 2D). Curdlan yield increases with urea concentration and higher agitation speed, indicating the importance of reducing urea and increasing agitation speed for enhancing the yield (Fig. 2E). On the other hand, lower agitation speed with lower KH_2_PO_4_ seemed to reduce the yield of curdlan (Fig. 2F). Overall, it can be deduced that a higher agitation speed of about 200 rpm and lower urea concentration of around 0.1% (w/v) is essential for improving curdlan production of *P. megaterium.*

The validation was performed employing the optimized parameter values as determined by the model: sucrose 20%, urea 0.1%, KH_2_PO_4_ 0.02%, agitation speed 250 rpm. Based on these values, the yield obtained was 0.46 g/L (predicted 0.70 g/L). The curdlan yield increased by 1.3-fold from the OFAT optimized conditions (0.35 g/L) in our previous study^24^ to the CCD optimized conditions (0.46 g/L). A significant increase in curdlan yield was observed from the unoptimized conditions (0.15 g/L) to CCD optimized conditions (0.46 g/L), representing a 3-fold increase in yield.

### Extraction of curdlan from the fermentation media

**Fig. 3:**
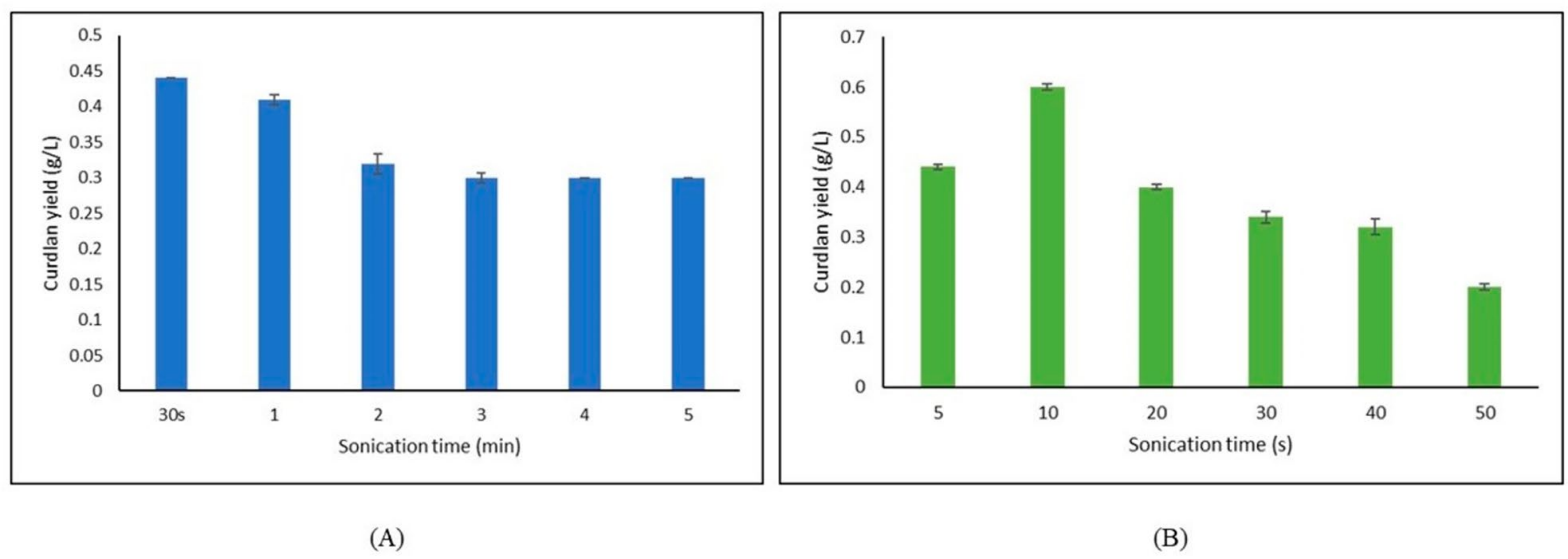
Effect of ultrasonication on curdlan yield (**A**) Bath sonication; (**B**) Probe sonication.

The conventional method of extraction with NaOH and HCl yielded 0.46 g/L curdlan under optimized conditions. To further enhance the extraction process, bath sonication was carried out at different time intervals viz., 30s, 1, 2, 3, 4, 5 min. 30 s gave the highest yield of curdlan at 0.44 g/L amongst other time intervals as shown in Fig. 3A. In the case of probe sonication, time intervals were 5, 10, 20, 30, 40, 50 s. 10 s probe sonication gave the maximum yield of curdlan at 0.60 g/L (Fig. 3B). Hence, probe sonication of 10 s was selected for further work. The energy density is lower in bath sonication as compared to probe sonication, thereby requiring higher sonication duration for bath sonication. Therefore, time was set differently between bath and probe sonication. A higher duration of probe sonication could have caused cell disruption, and hence was avoided.

The decline in curdlan yield following the optimal duration of sonication may be attributed to polymer size and effect of sonication time. Curdlan is a linear, high molecular weight biopolymer. Ultrasound frequency is more intensely absorbed by polymers with high molecular weights compared to those with lower molecular weights. Linear polymer chains may undergo easier chain degradation compared to branched or cross-linked polymers. During sonication, molecular degradation can occur randomly along the chain, with the extent of degradation being influenced by the duration of sonication^33^.

#### Optimization of extraction parameters by OFAT method

To improve the yield of curdlan, the OFAT method of optimization was employed for the extraction process. The primary benefit of utilizing the OFAT approach lies in its simplicity and convenience in conducting experiments. It is an effective tool to study the effect of different factors on product yield^28^.

**Fig. 4:**
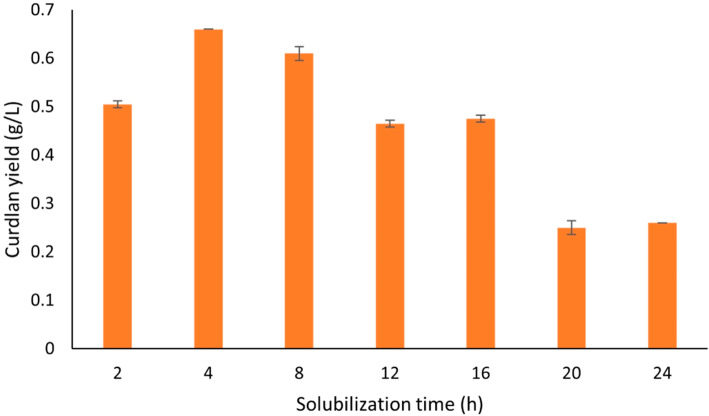
Effect of solubilization time (h) on curdlan yield (g/L).

Probe sonication proved to be better than bath sonication for improving the yield of curdlan (Fig. 3). The probable reason for this could be that curdlan being a cell-bound biopolymer, it gets detached more efficiently when subjected to higher energy ultrasonic waves^26^. Therefore, probe sonication was kept constant at 10 s along with NaOH concentration (2 N). The solubilization time was varied from 2 to 24 h. Notably, 4 h was found to be the most suitable solubilization time with the maximum yield of curdlan at 0.66 g/L as shown in Fig. 4.

**Fig. 5:**
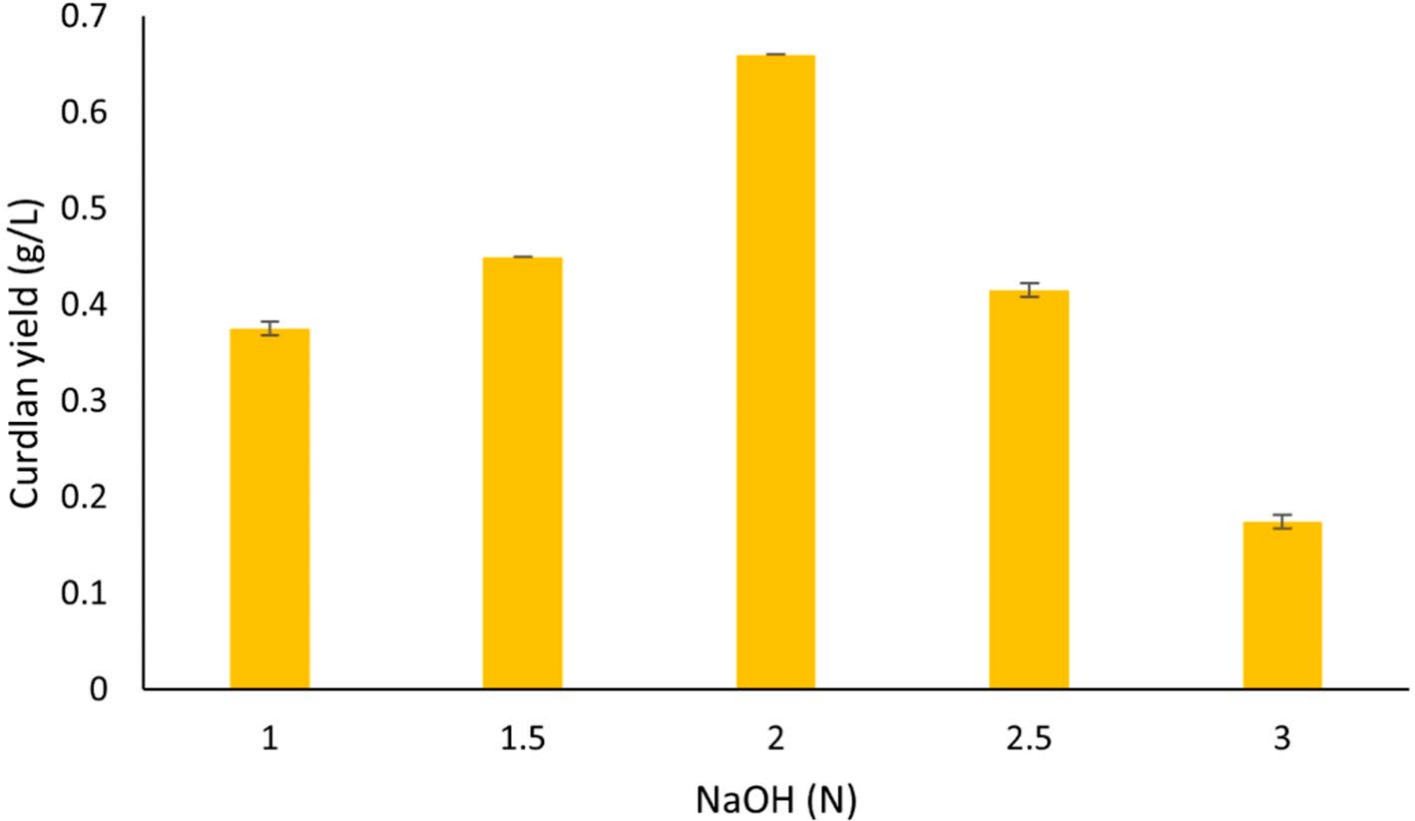
Effect of NaOH concentration (N) on curdlan yield (g/L).

The concentration of NaOH (2 N) was varied when the sonication and solubilization time was kept constant. It was observed that the optimal concentration of solubilization remained at 2 N NaOH (Fig. 5). The yield of curdlan began to decline with increased concentrations of NaOH after the optimal concentration of 2 N, following a similar trend. This could be due to the higher concentrations of NaOH which affect the hydrogen bonds in the structure. It is possible that the bonds undergo cleaving or degradation to some extent resulting in lower yield with increasing concentrations. A similar finding of bacterial cellulose films was reported where treatment with higher concentrations of NaOH resulted in the inter and intra-hydrogen bonds being cleaved in the cellulose films^34^.

#### Response surface methodology (RSM) by Central composite design (CCD) for extraction of curdlan

From the OFAT experiments, NaOH concentration, sonication and solubilization time were selected as 2 N, 10 s and 4 h, respectively. Based on this, CCD was carried out to further optimize and study the interaction effects of the factors in recovery of curdlan. The results of the experimental curdlan production by three factors are shown in Table 5. The maximum yield of curdlan production was found to be 0.67 g/L from run number 13 where the parameters were NaOH concentration 2 N, solubilization time 2 h, and sonication time 15 s. The minimal variation among the central points suggests that the experimental data exhibits notable reproducibility. The fitness of the model was given by ANOVA as shown in Table 6.

**Table 5:** Central composite design for Ultrasound-assisted extraction of curdlan with observed and predicted data.

Run no.	A: NaOH (*N*)	B: Solubilization time (h)	C: Sonication time (s)	Curdlan yield (g/L) Actual	Curdlan yield (g/L) Predicted
1	1	6	15	0.34	0.35
2	1.5	4	10	0.21	0.25
3	3	6	15	0.22	0.24
4	2	6	15	0.30	0.29
5	2.5	4	20	0.36	0.38
6	2	6	15	0.28	0.29
7	2	6	15	0.31	0.29
8	2.5	4	10	0.39	0.36
9	2	6	25	0.44	0.47
10	2	6	15	0.27	0.29
11	2	10	15	0.50	0.56
12	2.5	8	20	0.35	0.26
13	2	2	15	0.67	0.64
14	2.5	8	10	0.45	0.44
15	1.5	8	10	0.38	0.31
16	2	6	5	0.28	0.28
17	1.5	8	20	0.50	0.48
18	2	6	15	0.26	0.29
19	1.5	4	20	0.67	0.63
20	2	6	15	0.28	0.29

**Table 6:** ANOVA for curdlan yield with ultrasound-assisted extraction.

Source	Sum of squares	df	Mean square	F-value	*p*-value
Model	0.3025	9	0.0336	12.30	0.0003^*^
A-NaOH	0.0127	1	0.0127	4.63	0.0569
B-Solubilization time	0.0053	1	0.0053	1.92	0.1956
C-Sonication time	0.0371	1	0.0371	13.56	0.0042^*^
AB	0.0003	1	0.0003	0.1143	0.7423
AC	0.0630	1	0.0630	23.06	0.0007^*^
BC	0.0210	1	0.0210	7.69	0.0197^*^
A^2^	0.0002	1	0.0002	0.0575	0.8153
B^2^	0.1559	1	0.1559	57.05	< 0.0001^*^
C^2^	0.0127	1	0.0127	4.66	0.0563
Residual	0.0273	10	0.0027		
Lack of fit	0.0256	5	0.0051	14.77	0.0051^*^
Pure error	0.0017	5	0.0003		
Cor Total	0.3298	19			

*Represents statistically significant at 95% probability.

The F value of the model is 12.30 as shown in Table 6 which indicates that the model is significant. F value of lack of fit could be elevated due to noise. The pure error is remarkably low indicating high reproducibility of the acquired data. The coefficient of variation (C.V. %) was 14.02, reflecting good reproducibility (C.V. % <10% denotes excellent, 10–20% denotes good, 20–30% denotes adequate, and > 30% denotes poor reproducibility^29^), and the adequate precision value of 10.75 signified an adequate signal, as values above 4 are preferred. At a confidence level of 95% and *p* < 0.05, the linear term C in the current model is statistically significant. Among the two-way interactions, the AC, BC terms are significant and among the squared interactions, B^2^ is significant. The R^2^ for the correlation between the predicted and observed values for curdlan yield was 0.91. The model can explain up to 91% variation in the response. The model was modified by removing the insignificant terms. However, since the fitness of the model was better in the current model, hence the model is retained (Eq. 2). The results of the modified model are shown in the supplementary files for further information (Table S2).

The regression equation for the model in coded units is given in Eq. 2:

2 $$\begin{aligned} {\text{Y}}_{{({\text{g}}/{\text{L}})}} : & {\text{ }}0.{\text{29}}00{\text{ }} - {\text{ }}0.0{\text{281A }} - {\text{ }}0.0{\text{181B }} + {\text{ }}0.0{\text{481C }} + {\text{ }}0.00{\text{62AB }} \\ & \; - {\text{ }}0.0{\text{888AC }} - {\text{ }}0.0{\text{513BC }} + {\text{ }}0.00{\text{25A}}^{{\text{2}}} + {\text{ }}0.0{\text{787B}}^{{\text{2}}} + {\text{ }}0.0{\text{225C}}^{{\text{2}}} ~ \\ \end{aligned}$$

**Fig. 6:**
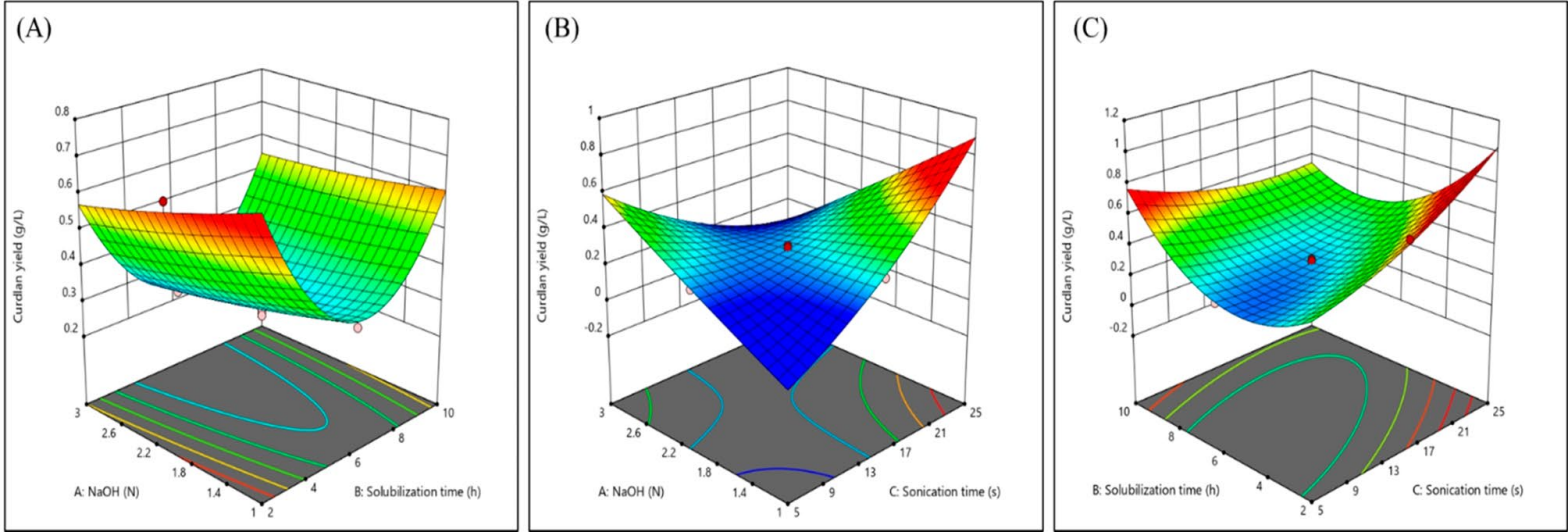
Response surface plot showing effect of different extraction parameters on curdlan yield. (**A**) NaOH and solubilization time; (**B**) NaOH and sonication time; (**C**) solubilization and sonication time. (Generated by Design-Expert software, version 9)

Where Y is the curdlan yield and A, B, C are NaOH concentration, solubilization time, sonication time, respectively. 3-D surface plots of curdlan yield and the factors were generated (Fig. 6) illustrating the dependency of curdlan yield on these factors. With lower NaOH concentrations and decreasing solubilization time, the curdlan yield seemed to improve (Fig. 6A). This is probably because of the degrading effect that NaOH has on polymer chains at higher concentrations. With increasing sonication time and decreasing NaOH concentrations, curdlan yield increases (Fig. 6B). It is known that low intensity ultrasonication or ultrasonication at very small-time intervals can lead to mass transfer^35^. In this case, it might facilitate better solubilization by homogenizing the cell-bound curdlan into the NaOH solution thereby increasing its yield. Curdlan yield increases when there is slight increase in sonication time and decrease in solubilization time (Fig. 6C). Overall, it is evident that recovery of curdlan is enhanced at a combination of lower NaOH concentrations, lesser solubilization time and longer sonication time. The novelty of this study is the curdlan extraction involving ultrasonication and its optimization using CCD.

This innovative approach aims to improve yield of curdlan by leveraging the mechanical effects of ultrasonication to enhance the solubilization process. This step distinguishes this study from previous methodologies, such as acid-alkali neutralization for precipitation, potentially setting a new standard for curdlan recovery.

Validation of the model was carried out using the optimized values of the factors as given by the software: NaOH concentration 1 N, solubilization time 2 h, sonication time 25 s. Using these values, the yield increased to 0.70 g/L (predicted 0.92 g/L). The curdlan yield increased by 1.5-fold from the CCD optimized conditions for media and process parameters (0.46 g/L) to the CCD optimized ultrasonication conditions (0.70 g/L). A notable increase in curdlan yield was observed from the unoptimized conditions (0.15 g/L) to CCD optimized conditions of extraction (0.70 g/L), representing a 4.6-fold increase in yield.

### Nuclear magnetic resonance (NMR) of curdlan

**Fig. 7:**
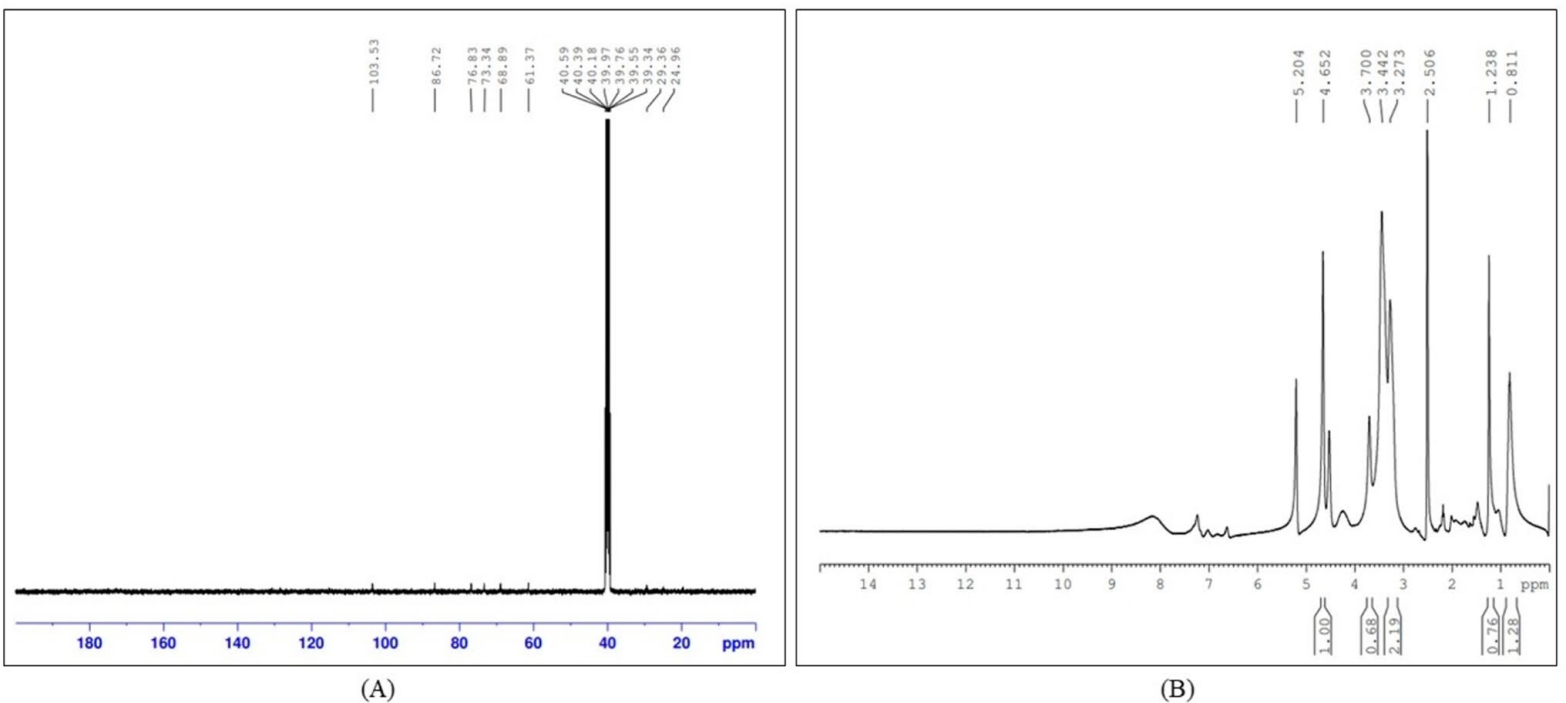
Nuclear magnetic resonance (NMR) spectra (**A**)^13^C NMR (**B**)^1^H NMR.

The NMR spectra as given in Fig. 7 showed characteristic peaks of curdlan thereby verifying the structure of curdlan. The^1^H NMR spectra showed 7 protons between 3.0 and 4.5 ppm whereas the^13^C NMR spectra showed 6 resonances ~ 103.8 (C-1), 74.2 (C-2), 87.32 (C-3), 68.82 (C-4), 77.2 (C-5), 61.25 (C-6) ppm representing the β-(1,3)-D-glucan backbone. The NMR spectra obtained following curdlan extraction using ultrasonication revealed no significant changes in its structural properties. The resonances obtained were consistent with those reported in previous studies, indicating that ultrasonication did not affect the inherent characteristics of curdlan^14,36–38^. Fourier transform infrared spectroscopy (FTIR), scanning electron microscopy (SEM), and thermogravimetric analysis (TGA) of curdlan from our previous results^24^ revealed characteristic results consistent with those obtained from NMR spectroscopy, comprehensively confirming the biopolymer as curdlan.

## Conclusion

In this work, the fermentative yield of curdlan was enhanced by optimizing the media and extraction method. The optimized fermentation media consisted of 20% sucrose, 0.1% urea, 0.02% KH_2_PO_4_ at agitation speed of 250 rpm. The results revealed that a higher agitation speed and lower urea concentration positively impacted the yield of curdlan. A novel method of curdlan extraction was employed wherein ultrasonication was introduced for improving curdlan yield. A significant increase was observed in the yield when probe sonication of 10 s was carried out after the addition of NaOH. The optimization of the extraction process parameters, namely, NaOH concentration, solubilization time, and sonication time were conducted using both OFAT and CCD to enhance the yield of curdlan. The optimized conditions of extraction parameters after CCD were NaOH concentration, 1 N; solubilization time, 2 h; and sonication time, 25 s. It was also noted that the extraction time was significantly reduced which, in turn, decreased the overall process time from production to recovery, providing an added advantage. Overall, the yield has increased by 1.5-fold from the conventional extraction method (0.46 g/L) to the CCD extraction optimized conditions (0.70 g/L). This is 4.6-fold increase in curdlan yield when compared to unoptimized fermentation process conditions. Curdlan extracted from *P. megaterium* was characterized by NMR where it has shown the characteristic structure of curdlan as reported in other studies. Further characterization of curdlan can be carried out such as estimation of molecular weight and Polydispersity. Chemical modification of curdlan to enhance its properties for their possible applications in food sector and drug delivery can also be taken up for further studies. In addition, the scale up studies using ultrasonication assisted extraction can also be explored.

## Electronic Supplementary Material

Below is the link to the electronic supplementary material.

Supplementary Material 1

## Data Availability

Data can be made available from Subbalaxmi Selvaraj upon reasonable request.

## References

[1] Wan, J., Shao, Z., Jiang, D., Gao, H. & Yang, X. Curdlan production from cassava starch hydrolysates by Agrobacterium sp. DH-2. *Bioprocess. Biosyst. Eng.* **45**, 969–979 (2022). 35312865 10.1007/s00449-022-02718-8

[2] Li, Y., Wan, J., Gao, H. & Yang, X. Production of curdlan by agrobacterium sp. DH-2 using sugarcane molasses-based medium. *J. Polym. Environ.* **31**, 4382–4392 (2023).

[3] Zhang, R. & Edgar, K. J. Properties, chemistry, and applications of the bioactive polysaccharide curdlan. *Biomacromolecules* **15**, 1079–1096 (2014). 24552241 10.1021/bm500038g

[4] Ben Salah, R. et al. Fermentation of date palm juice by curdlan gum production from Rhizobium radiobacter ATCC 6466™: Purification, rheological and physico-chemical characterization. *LWT - Food Sci. Technol.* **44**, 1026–1034 (2011).

[5] Gummadi, S. N. & Kumar, K. Production of extracellular water insoluble β-1,3-glucan (Curdlan) from Bacillus sp. SNC07. *Biotechnol. Bioprocess. Eng.* **10**, 546–551 (2005).

[6] Kenyon, W. J., Esch, S. W. & Buller, C. S. The curdlan-type exopolysaccharide produced by cellulomonas flavigena KU forms part of an extracellular glycocalyx involved in cellulose degradation. *Antonie Van Leeuwenhoek*. **87**, 143–148 (2005). 15723175 10.1007/s10482-004-2346-4

[7] Mamaril, J. C., Paner, E. T. & Palacpac, E. S. The production of gel-forming polysaccharides by Rhizobium sp. and curdlan by a mutant cultured in coconut water. *Trans. Nat. Acad. Sci. Tech.* **10**, 339–349 (1988).

[8] Yu, X. et al. CrdR function in a curdlan-producing Agrobacterium sp. ATCC31749 strain. *BMC Microbiol.* **15**, 1–10 (2015). 25880528 10.1186/s12866-015-0356-1 PMC4327974

[9] Aquinas, N., Bhat, M. & Selvaraj, S. R. A review presenting production, characterization, and applications of biopolymer curdlan in food and pharmaceutical sectors. *Polymer Bulletin* **79**, 6905–6927 at (2022). 10.1007/s00289-021-03860-1

[10] Zhang, C. et al. Ultrasonic treatment combined with curdlan improves the gelation properties of low-salt Nemipterus virgatus surimi. *Int. J. Biol. Macromol.* **248**, (2023). 10.1016/j.ijbiomac.2023.125899 37479203

[11] Rafigh, S. M., Yazdi, A. V., Vossoughi, M., Safekordi, A. A. & Ardjmand, M. Optimization of culture medium and modeling of curdlan production from Paenibacillus polymyxa by RSM and ANN. *Int. J. Biol. Macromol.* **70**, 463–473 (2014). 25062991 10.1016/j.ijbiomac.2014.07.034

[12] Martinez, C. O. et al. Characterization of curdlan produced by Agrobacterium sp. IFO 13140 cells immobilized in a loofa sponge matrix, and application of this biopolymer in the development of functional yogurt. *J. Sci. Food Agric.* **96**, 2410–2417 (2016). 26219432 10.1002/jsfa.7357

[13] Yuan, M., Fu, G., Sun, Y. & Zhang, D. Biosynthesis and applications of curdlan. *Carbohydr. Polym.* **273**, (2021). 10.1016/j.carbpol.2021.118597 34560997

[14] Shih, I. L., Yu, J. Y., Hsieh, C. & Wu, J. Y. Production and characterization of curdlan by Agrobacterium sp. *Biochem. Eng. J.* **43**, 33–40 (2009).

[15] West, T. P. Production of the polysaccharide curdlan by agrobacterium species on processing coproducts and plant lignocellulosic hydrolysates. *Fermentation* **6**, 1–10 (2020).

[16] Adekunle, A., Ukaigwe, S., Bezerra dos Santos, A. & Iorhemen, O. T. Potential for curdlan recovery from aerobic granular sludge wastewater treatment systems – A review. *Chemosphere* **142504**10.1016/j.chemosphere.2024.142504 (2024). 10.1016/j.chemosphere.2024.142504 38825243

[17] Aquinas, N., Bhat, M. & Selvaraj, S. R. Curdlan based hydrogels. in *Polysaccharide hydrogels for drug delivery and regenerative medicine* (eds. Giri, T. K., Ghosh, B. & Badwaik, H.) 203–212 (Elsevier, 2023).

[18] Lee, I. Y. et al. Production of curdlan using sucrose or sugar cane molasses by two-step fed-batch cultivation of Agrobacterium species. *J. Ind. Microbiol. Biotechnol.* **18**, 255–259 (1997).

[19] Anane, R. F. et al. Improved curdlan production with discarded bottom parts of Asparagus spear. *Microb. Cell. Fact.* **16**, 1–8 (2017). 28388915 10.1186/s12934-017-0671-3 PMC5384130

[20] Kurian, N. S. & Das, B. Comparative analysis of various extraction processes based on economy, eco-friendly, purity and recovery of polyhydroxyalkanoate: A review. *International Journal of Biological Macromolecules* vol. 183 1881–1890 at (2021). 10.1016/j.ijbiomac.2021.06.007 10.1016/j.ijbiomac.2021.06.007 34090850

[21] Momen, S., Alavi, F. & Aider, M. Alkali-mediated treatments for extraction and functional modification of proteins: Critical and application review. *Trends in Food Science and Technology* vol. 110 778–797 at (2021). 10.1016/j.tifs.2021.02.052

[22] Cui, J. D. & Qiu, J. Q. Production of extracellular water-insoluble polysaccharide from Pseudomonas sp. *J. Agric. Food Chem.* **60**, 4865–4871 (2012). 22533491 10.1021/jf3006273

[23] Yang, H. et al. Optimization and modeling of curdlan production under multi-physiological-parameters process control by agrobacterium radiobacter mutant A-15 at high initial glucose. *Biotechnol. Bioprocess. Eng.* **26**, 1012–1022 (2021).

[24] Aquinas, N., Bhat, R. M. & Selvaraj, S. Submerged fermentation and kinetics of newly isolated priestia megaterium for the production of biopolymer curdlan. *J. Polym. Environ.*10.1007/s10924-024-03224-6 (2024).

[25] Prakash, S. et al. Optimization and production of curdlan gum using Bacillus cereus PR3 isolated from rhizosphere of leguminous plant. *Prep Biochem. Biotechnol.* **48**, 408–418 (2018). 29561223 10.1080/10826068.2018.1451886

[26] Li, J. et al. Ultrasound-assisted extraction and properties of polysaccharide from Ginkgo biloba leaves. *Ultrason. Sonochem* **93**, (2023). 10.1016/j.ultsonch.2023.106295 PMC9852606 36638652

[27] Yin, D. et al. Structural properties and antioxidant activity of polysaccharides extracted from Laminaria japonica using various methods. *Process. Biochem.* **111**, 201–209 (2021).

[28] Singh, V. et al. Strategies for fermentation medium optimization: An in-depth review. *Front. Microbiol.* **7**, (2017). 10.3389/fmicb.2016.02087 PMC5216682 28111566

[29] Aronhime, S. et al. DCE-MRI of the liver: Effect of linear and nonlinear conversions on hepatic perfusion quantification and reproducibility. *J. Magn. Reson. Imaging* **40**, 90–98 (2014). 24923476 10.1002/jmri.24341 PMC4058642

[30] Das, D., Selvaraj, R. & Ramananda Bhat, M. Optimization of inulinase production by a newly isolated strain Aspergillus flavus var. flavus by solid state fermentation of Saccharum arundinaceum. *Biocatal. Agric. Biotechnol.* **22**, (2019).

[31] Shet, V. B. et al. Optimization of reducing sugars production from agro-residue coconut leaflets using autoclave-assisted HCl hydrolysis with response surface methodology. *Agric. Nat. Resour.* **52**, 280–284 (2018).

[32] Kim, M. K. et al. Residual phosphate concentration under nitrogen-limiting conditions regulates curdlan production in agrobacterium species. *J. Ind. Microbiol. Biotechnol.* **25** (2000). https://academic.oup.com/jimb/article/25/4/180/5990433

[33] Biswas, S. & Rashid, T. U. Effect of ultrasound on the physical properties and processing of major biopolymers—a review. *Soft Matter* **18**, 8367–8383 (2022). 36321472 10.1039/d2sm01339h

[34] Suryanto, H. et al. Institute of Physics Publishing,. The mechanical strength and morphology of bacterial cellulose films: The effect of NaOH concentration. in *IOP Conference Series: Materials Science and Engineering* vol. 515 (2019).

[35] Behzadnia, A., Moosavi-Nasab, M., Ojha, S. & Tiwari, B. K. Exploitation of ultrasound technique for enhancement of microbial metabolites production. *Molecules* **25** at (2020). 10.3390/MOLECULES25225473 10.3390/molecules25225473 PMC7700470 33238482

[36] Kalyanasundaram, G. T., Doble, M. & Gummadi, S. N. Production and downstream processing of (1→3)-β- D-glucan from mutant strain of Agrobacterium sp. ATCC 31750. *AMB Express* **2**, 1–10 (2012). 22681895 10.1186/2191-0855-2-31 PMC3573891

[37] Rofeal, M., Abdelmalek, F., Pietrasik, J. & Steinbüchel, A. Sustainable curdlan biosynthesis by Rahnella variigena ICRI91 via alkaline hydrolysis of Musa sapientum peels and its edible, active and modified hydrogel for Quercetin controlled release. *Int. J. Biol. Macromol.* **225**, 416–429 (2023). 36375664 10.1016/j.ijbiomac.2022.11.080

[38] Wang, X. Y. Z., Dong, J. J., Xu, G. C., Han, R. Z. & Ni, Y. Enhanced curdlan production with nitrogen feeding during polysaccharide synthesis by Rhizobium radiobacter. *Carbohydr. Polym.* **150**, 385–391 (2016). 27312649 10.1016/j.carbpol.2016.05.036

