# Supplementary material for: Optimization of curdlan production and ultrasound assisted extraction processes from Priestia megaterium
Source: Sci Rep. 2024 Nov 4;14:26709. doi: 10.1038/s41598-024-77880-y (PMC11535322; doi:10.1038/s41598-024-77880-y)
Supplement: Supplementary file 1 — Supplementary Material 1 [file 41598_2024_77880_MOESM1_ESM.docx]

**Optimization of Curdlan Production and Ultrasound assisted Extraction Processes from *Priestia megaterium***

**Natasha Aquinas^1^, Ramananda Bhat M^1*^, Subbalaxmi Selvaraj^1*^**

**Supplementary material**

Table S1: ANOVA for optimization of fermentation media for Curdlan yield

| Source | Sum of squares | df | Mean square | F-value | p-value |
| --- | --- | --- | --- | --- | --- |
| Model | 0.3216 | 10 | 0.0326 | 29.38 | <0.0001^*^ |
| A-Sucrose | 0.0170 | 1 | 0.0170 | 15.33 | 0.0009^*^ |
| B-Urea | 0.0045 | 1 | 0.0045 | 4.06 | 0.0582 |
| C-KH_2_PO_4_ | 0.0117 | 1 | 0.0117 | 10.50 | 0.0043^*^ |
| D-Agitation | 0.2033 | 1 | 0.2033 | 183.17 | <0.0001^*^ |
| AC | 0.0081 | 1 | 0.0081 | 7.26 | 0.0144^*^ |
| AD | 0.0421 | 1 | 0.0421 | 37.95 | <0.0001^*^ |
| BC | 0.0076 | 1 | 0.0076 | 6.86 | 0.0169^*^ |
| A^2^ | 0.0088 | 1 | 0.0088 | 7.96 | 0.0109^*^ |
| B^2^ | 0.0088 | 1 | 0.0088 | 7.96 | 0.0109^*^ |
| C^2^ | 0.0109 | 1 | 0.0109 | 9.83 | 0.0054 |
| Residual | 0.0211 | 19 | 0.0011 |  |  |
| Lack of fit | 0.0206 | 14 | 0.0015 | 13.77 | 0.0045 |
| Pure error | 0.0005 | 5 | 0.0001 |  |  |
| Cor Total | 0.3472 | 29 |  |  |  |

*represents statistically significant at 95% probability; R^2^ = 93.9, adjusted R^2^ = 90.7, adequate precision = 18.54, C.V.% 19.95

The modified regression equation for the model in coded units is given in Equation 1:

Y _(g/L) =_ 0.1797 + 0.0266A - 0.0137B + 0.0220C + 0.0920D - 0.0224AC + 0.0513AD + 0.0218BC - 0.0178A^2^ - 0.0178B^2^ + 0.0197C^2^ (1)

Table S2: ANOVA for optimization of ultrasound-assisted extraction of curdlan yield

| Source | Sum of squares | df | Mean square | F-value | p-value |
| --- | --- | --- | --- | --- | --- |
| Model | 0.3020 | 7 | 0.0431 | 18.62 | <0.0001^*^ |
| A-NaOH | 0.0127 | 1 | 0.0127 | 5.46 | 0.0376^*^ |
| B-Solubilization time | 0.0053 | 1 | 0.0053 | 2.27 | 0.1579^*^ |
| C-Sonication time | 0.0371 | 1 | 0.0371 | 15.99 | 0.0018^*^ |
| AC | 0.0630 | 1 | 0.0630 | 27.20 | 0.0002^*^ |
| BC | 0.0210 | 1 | 0.0210 | 9.07 | 0.0108^*^ |
| B^2^ | 0.1612 | 1 | 0.1612 | 69.59 | <0.0001^*^ |
| C^2^ | 0.0127 | 1 | 0.0127 | 5.49 | 0.0372^*^ |
| Residual | 0.0278 | 12 | 0.023 |  |  |
| Lack of fit | 0.0261 | 7 | 0.0037 | 10.74 | 0.0094^*^ |
| Pure error | 0.0017 | 5 | 0.0003 |  |  |
| Cor Total | 0.3298 | 19 |  |  |  |

*represents statistically significant at 95% probability; R^2^ = 91.5, adjusted R^2^ = 86.6, adequate precision = 13.31, C.V.% = 12.90

The modified regression equation for the model in coded units is given in Equation 2:

Y _(g/L)_: 0.2929 - 0.0281A - 0.0181B + 0.0481C - 0.0888AC - 0.0513BC + 0.0782B^2^ + 0.0220C^2^ (2)
